# The plasma membrane calcium ATPase 4 does not influence parasite levels but partially promotes experimental cerebral malaria during murine blood stage malaria

**DOI:** 10.1186/s12936-021-03832-w

**Published:** 2021-07-02

**Authors:** Ana Villegas-Mendez, Nicholas Stafford, Michael J. Haley, Normalita Eka Pravitasari, Florence Baudoin, Adnan Ali, Puji Budi Setia Asih, Josephine E. Siregar, Esther Baena, Din Syafruddin, Kevin N. Couper, Delvac Oceandy

**Affiliations:** 1 The Lydia Becker Institute of Immunology and Inflammation, Faculty of Biology, Medicine and Health, University of Manchester, Manchester, M13 9PT UK; 2 Division of Cardiovascular Sciences, Faculty of Biology, Medicine and Health, Manchester Academic Health Science Centre, The University of Manchester, Manchester, M13 9PT UK; 3 Eijkman Institute for Molecular Biology, Jakarta, 10430 Indonesia; 4 Cancer Research UK Manchester Institute, The University of Manchester, Alderley Park, Manchester, SK10 4TG UK; 5 Present Address: Division of Cancer Sciences, The University of Manchester, Manchester, UK

**Keywords:** PMCA4, Malaria, Knockout mice, Plasmodium, Cerebral malaria, Red blood cell

## Abstract

**Background:**

Recent genome wide analysis studies have identified a strong association between single nucleotide variations within the human *ATP2B4* gene and susceptibility to severe malaria. The *ATP2B4* gene encodes the plasma membrane calcium ATPase 4 (PMCA4), which is responsible for controlling the physiological level of intracellular calcium in many cell types, including red blood cells (RBCs). It is, therefore, postulated that genetic differences in the activity or expression level of PMCA4 alters intracellular Ca^2+^ levels and affects RBC hydration, modulating the invasion and growth of the *Plasmodium* parasite within its target host cell.

**Methods:**

In this study the course of three different *Plasmodium* spp. infections were examined in mice with systemic knockout of *Pmca4* expression.

**Results:**

Ablation of PMCA4 reduced the size of RBCs and their haemoglobin content but did not affect RBC maturation and reticulocyte count. Surprisingly, knockout of PMCA4 did not significantly alter peripheral parasite burdens or the dynamics of blood stage *Plasmodium chabaudi* infection or reticulocyte-restricted *Plasmodium yoelii* infection. Interestingly, although ablation of PMCA4 did not affect peripheral parasite levels during *Plasmodium berghei* infection, it did promote slight protection against experimental cerebral malaria, associated with a minor reduction in antigen-experienced T cell accumulation in the brain.

**Conclusions:**

The finding suggests that PMCA4 may play a minor role in the development of severe malarial complications, but that this appears independent of direct effects on parasite invasion, growth or survival within RBCs.

**Supplementary Information:**

The online version contains supplementary material available at 10.1186/s12936-021-03832-w.

## Background

Malaria is a major public health problem in developing countries, primarily in sub-Saharan Africa and Asia [1]. There were 229 million clinical cases of malaria resulting in approximately 409,000 deaths reported in 2019, making malaria one of the most prevalent and deadly infectious diseases in the world [1]. Despite significant efforts to reduce the incidence of malaria in the past decade, progress in controlling and eliminating malaria has stalled in the last 2–3 years, with the number of infections beginning to rise in a number of countries [1, 2]. Moreover, there is increasing evidence that *Plasmodium falciparum* parasites, the causative agent of malaria, are developing resistance to a number of front-line anti-malarial drugs (such as artemisinin), which has the potential to severely impact malaria control in the future [3, 4]. Consequently, there is still an urgent need to develop new treatments for malaria.

Understanding the critical host pathways that control the susceptibility and resistance of individuals to malaria in endemic countries may afford novel strategies to treat severe malaria. A recent human genome wide association study (GWAS) in Kenya identified single nucleotide polymorphisms (SNPs) in the human plasma membrane calcium ATPase 4 (PMCA4) gene (also called *ATP2B4*) to have a very strong association with resistance and severity of malaria infection [5]. This study confirmed results in two earlier GWAS (one in Ghana and one multi-site across Africa, Asia and Oceania) identifying *ATP2B4* as a significant resistance loci for severe malaria [6, 7]. The reproducibility in identification of *ATP2B4* across different populations and studies suggests a major role for the gene product of *ATP2B4* in modulating the severity of *Plasmodium* infection.

PMCAs are ATP-driven and calmodulin-dependent calcium pumps that eject calcium from the cell cytoplasm to the extra-cellular compartment [8]. There are four PMCA isoforms that have been identified (PMCA1-4). It is believed that PMCA1 and PMCA4 are ubiquitously expressed whereas PMCA2 and PMCA3 expressions are confined to specific tissues [9]. Importantly, PMCA4 expression can be detected in red blood cells (RBC), the target cell of *Plasmodium* parasites during blood-stage malaria. In RBC, PMCA is the only active calcium extrusion pump required to maintain intracellular calcium in the 20–50 nM range [10]. Thus, inhibition of PMCA4 activity significantly elevated intracellular calcium in RBCs [10, 11]. Notably, the growth defect of *Plasmodium* parasites in cold stored (4 °C) RBCs has been associated with inhibition of PMCA channel activity and increased intracellular Ca^2+^ concentrations [12]. The increase in intracellular Ca^2+^ concentrations can lead to RBC dehydration [13], and there is evidence that *P. falciparum* parasites are unable to invade and grow as efficiently in dehydrated RBCs [14]. Targeting and manipulating the activity of PMCA4 may, therefore, be a novel strategy for treatment of malaria.

In this study to directly assess the role of PMCA4 in modulating the course of blood stage malaria, mice with a systemic genetic knockout of the *Pmca4* gene (PMCA4^−/−^ mice) were infected with different species of murine *Plasmodium* parasites. Surprisingly, whilst abrogation of PMCA4 influences the morphology and biology of RBCs, it did not substantially affect peripheral parasite levels during *Plasmodium yoelii*, *Plasmodium chabaudi* or *Plasmodium berghei* infections. Inhibition of PMCA4 did, however, slightly increase the resistance of mice to experimental cerebral malaria (ECM). The results suggest that PMCA4 may have an effect on the development of severe malaria disease but raise important questions on the mechanisms involved.

## Methods

### Ethics statement

All animal work was approved following local ethical review by the University of Manchester (UoM) Animal Procedures and Ethics Committees and was performed in strict accordance with the U.K Home Office Animals (Scientific Procedures) Act 1986 (approved H.O Project Licence P8829D3B4).

### Mice and infections

PMCA4 global knock out (PMCA4^−/−^) mice and wild type (WT) littermates were used in this study. Generation of PMCA4^−/−^ mice was described in our previous publication [15]. Uninfected global PMCA4^−/−^ mice are generally normal, as defined by whole body phenotype analysis [16]. Mice were bred at the Biological Service Facility (BSF), University of Manchester. All mice were maintained in specific-pathogen free conditions in individually ventilated cages. The *Plasmodium chabaudi* used was the non-lethal AS strain obtained from Dr K.N. Brown (National Institute for Medical Research (NIMR), London) [17]. The *Plasmodium yoelii* NL strain was obtained from Dr Brian De Souza (UCL, London) [18], whereas the *Plasmodium berghei* ANKA used was the clone cl15cy1 as described in [19]. Depending upon the experiment, cryopreserved *P. chabaudi* AS, *P. yoelii* NL or *P. berghei* ANKA parasites were passaged once in C57BL/6 mice before experimental mice were infected via intravenous (i.v.) injection of 10^4^ parasitized red blood cells (pRBC) of one of the *Plasmodium* species. The course of infection was monitored by assessing peripheral parasitaemia via microscopic examination of Giemsa-stained thin blood smears, by calculating mouse weight on specified days of infection (compared to starting weight at initiation of experiment), and by quantifying erythrocyte numbers in peripheral blood (RBC /ml) by microscopy using a C-Chip disposable haemocytometer (Cambridge Bioscience). ECM was graded during *P. berghei* ANKA infection as previously described [20]: 1 = no signs; 2 = ruffled fur/and or abnormal posture; 3 = lethargy; 4 = reduced responsiveness to stimulation and/or ataxia and/or respiratory distress/hyperventilation; 5 = prostration and/or paralysis and/or convulsions. Stages 2 and 3 were classified as prodromal ECM and stages 4–5 were classified as ECM. *Plasmodium berghei* infected mice were euthanized when they reached stage 4/5. To examine the permeability of the blood brain barrier (an important event during ECM), mice were injected i.v. with 200 ml of 1% Evans blue (Sigma Aldrich, UK) on day 6 of *P. berghei* infection. After one hour, mice were culled and intra-cardiac whole-body perfusion with 15 ml of PBS was performed, following which brains were removed.

### Automated blood analysis

For peripheral blood analysis, mice were anesthetized using 2.5% isoflurane and blood was collected from the jugular vein. Blood samples were analysed within 6 h after collection using an automated blood analyser (Sysmex XT-2000iV) at room temperature. Blood analysis was carried out in 2 batches using a mouse profile.

### Western blot

For western blot analysis of erythrocyte PMCA expression, blood was collected from the jugular vein of PMCA4^−/−^ and WT mice under anaesthesia with 2.5% isoflurane, and heparinized. Blood was centrifuged at 1800*g* for 5 min and the plasma and buffy coat were discarded. Erythrocytes were then washed 4 times in twice their volume of 0.9% saline, with centrifugation for 5 min at 1200*g*. Packed erythrocytes were then lysed in 10 × their volume of RIPA buffer (containing 1% IGEPAL CA-630, 0.5% sodium deoxycholate, 0.1% SDS, 0.5 mM phenylmethylsulphonyl fluoride, 500 ng/ml Leupeptin, 1 mg/ml Aprotinin and 2.5 mg/ml Pepstatin A), and 10 µl of the resultant lysate was separated by SDS-PAGE and transferred to PVDF membranes (Millipore) using standard protocols.

Membranes were blocked in 3% BSA (for PMCA4 detection) or 3% non-fat milk (for PMCA1 detection) for 1 h at room temperature, before refrigerated incubation overnight with primary antibodies against PMCA4 (clone JA9-Abcam) or PMCA1 (clone F-10-Santa Cruz). Proteins were visualized the following day using enhanced chemiluminescence (GE Healthcare), after incubation with HRP-linked anti-mouse or anti-rabbit secondary antibody (Cell Signalling) on a ChemiDoc XRS Imaging System (Biorad). Membranes were then incubated with β-actin or GAPDH antibody (abcam) as loading control.

### Image stream

Heparinized blood obtained from the tail vein was surface stained for 25 min at 4 ^Ο^C with anti-Ter119 (Ter119—BioLegend), anti-CD44 (IM7—Thermofisher) and anti-CD71 (R17217—Thermofisher), before being washed and resuspended in PBS containing DAPI. Cells were analysed with an ImageStream X Mk II (Amnis) and data was analysed with IDEAS (Amnis).

### Analysis of RBCs morphology following *Plasmodium* infection

Images of Giemsa-stained thin blood smears from WT and PMCA4^−/−^ mice on days 7 and 9 of *P. chabaudi* and *P. yoelii* infection were uploaded to Image Pro Premier image analysis software. Automated classification of RBCs was performed with a lower size threshold of 3000 pixels (to exclude small cell fragments and circulating material) and a roundness threshold of < 1.2 (to exclude overlapping cell populations). A total of 38 fields of view from 3 mice per group per time point was analysed.

### Flow cytometry

Spleens were removed from infected and uninfected PMCA4^−/−^ and WT control mice and homogenized through a 70 µm cell sieve to create single cell suspensions (BD Biosciences). RBCs were lysed in the samples by addition of RBC lysing buffer (BD Biosciences), following which cells were washed and resuspended in HBSS containing 2% FCS (FACS buffer). Brains were isolated from mice after intra-cardial perfusion with PBS, chopped into small pieces using scissors, and passed through a 10 ml syringe, before being incubated on a tube roller at room temperature in FACS buffer with Collagenase (final concentration 1 mg/ml) (Sigma) for 45 min. The resultant brain cell suspensions were filtered through a 70 µm cell sieve, washed in FACS buffer, and layered on a 30% Percoll gradient and centrifuged at 2000*g* for 10 min. The supernatant was discarded and the cell pellet collected. RBCs were lysed in the samples by addition of RBC lysing buffer (BD Biosciences) and cells were washed and resuspended in FACS buffer. Absolute cell numbers were determined by microscopy using a haemocytometer and live/dead differentiation was performed using the trypan blue exclusion cell viability assay (Sigma).

Spleen and brain samples were surface stained for 20 min with anti-mouse CD4 (GK1.5—Thermofisher), anti-mouse CD8a (53–6.7—Thermofisher), anti-mouse CD11a (M17/4—Thermofisher), anti-mouse CD11b (M1/70—BioLegend), anti-mouse CD31 (390—BioLegend), anti-mouse CD45 (30-F11—Thermofisher), anti-mouse CD49d (R1-2—BioLegend), anti-mouse ICAM-1 (YN1/1.7.4—BioLegend), anti-mouse Ly6C (HK1.4—BioLegend) and anti-mouse Ly6G (1A8—BioLegend). Intracellular staining for granzyme B (GB11—BioLegend) was performed for 45 min, after treatment with Foxp3 fixation/permeabilization buffer (Thermofisher). Dead cells were identified and exluded from analysis using LIVE/DEAD® Fixable Blue Dead Cell Stain Kit (Life Technologies). Fluoresce minus one controls were used to set gates. Cells were analysed with a BD LSR II (Becton Dickinson) using BD FACSDiva software (Becton Dickinson). Data was analysed with FlowJo (Tree Star Inc.).

### Statistical analyses

Data was tested for normality using the Shapiro–Wilk test. Unpaired two tailed *t* test (for parametric data) or the Mann–Whitney *U* test (for non-parametric data) was used for comparison between two groups. One-way ANOVA, with Tukey post hoc analysis, or the Kruskal–Wallis test, with Dunn post hoc analysis, were used for comparisons between three or more groups, for parametric and non-parametric data, respectively. Survival data was analysed using the Mantel-Cox test. Results were considered as significantly different when *p* < 0.05.

## Results

### Effects of PMCA4 ablation on RBC size and maturation state

**Fig. 1:**
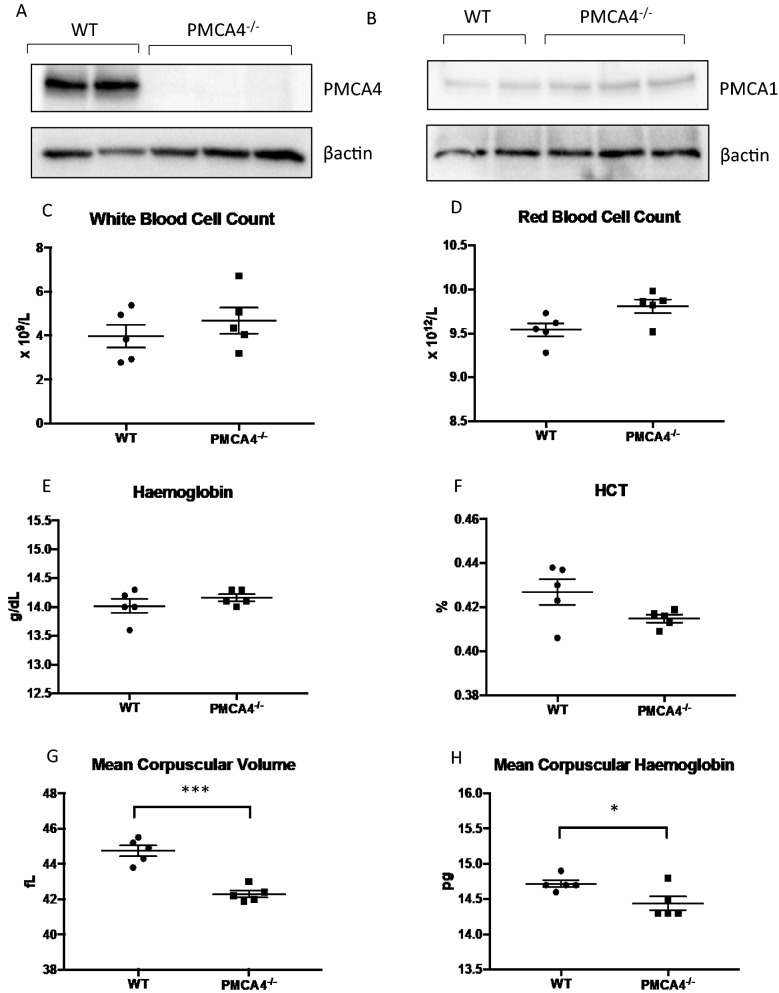
The effect of PMCA4 deletion on RBC properties. Blood was obtained from uninfected PMCA4^−/−^ and wild type (WT) littermates. **A** Western blot analysis examining **A** PMCA4 and **B** PMCA1 expression in whole blood cell lysates. **C**–**H** Whole blood examination by automated blood analyser investigating **C** total RBC count, **D** white blood cell counts, **E** haemoglobin level, **F** haematocrit, **G** mean corpuscular volume and **H** mean corpuscular haemoglobin level (n = 5 in each group, *P < 0.05, **P < 0.01)

To investigate how PMCA4 controls murine RBC biology, RBCs from mice with a global knockout of the *Pmca4* gene (generated via deletion of exon 2 of mouse *Pmca4* gene, which contains the start codon) were analysed [15]. Western blot analysis of whole blood lysates confirmed the complete ablation of PMCA4 expression in blood of PMCA4^−/−^ mice, compared with WT control mice (Fig. 1A). Importantly, PMCA1 was expressed at a low level in whole blood of WT mice, indicating that PMCA4 was the dominant PMCA on circulating blood cells (Fig. 1B). The level of PMCA1 was, however, slightly increased on whole blood lysates from PMCA4^−/−^ mice, potentially as a compensatory response (Fig. 1B).

Analysis using an automatic blood analyser system indicated that the RBCs of PMCA4^−/−^ mice were slightly smaller in size than RBCs of control mice as shown by the mean corpuscular volume (MCV) values. Moreover, RBCs of PMCA4^−/−^ mice also had lower mean corpuscular haemoglobin (MCH) values than RBCs from control mice, suggesting that the cells in the PMCA4^−/−^ mice had less haemoglobin per RBC. Other RBC parameters such as RBC total count, haemoglobin level and haematocrit were not altered in PMCA4^−/−^ mice compared with control mice, and total white blood cell count was also not affected by PMCA4 deletion (Fig. 1C–H).

**Fig. 2:**
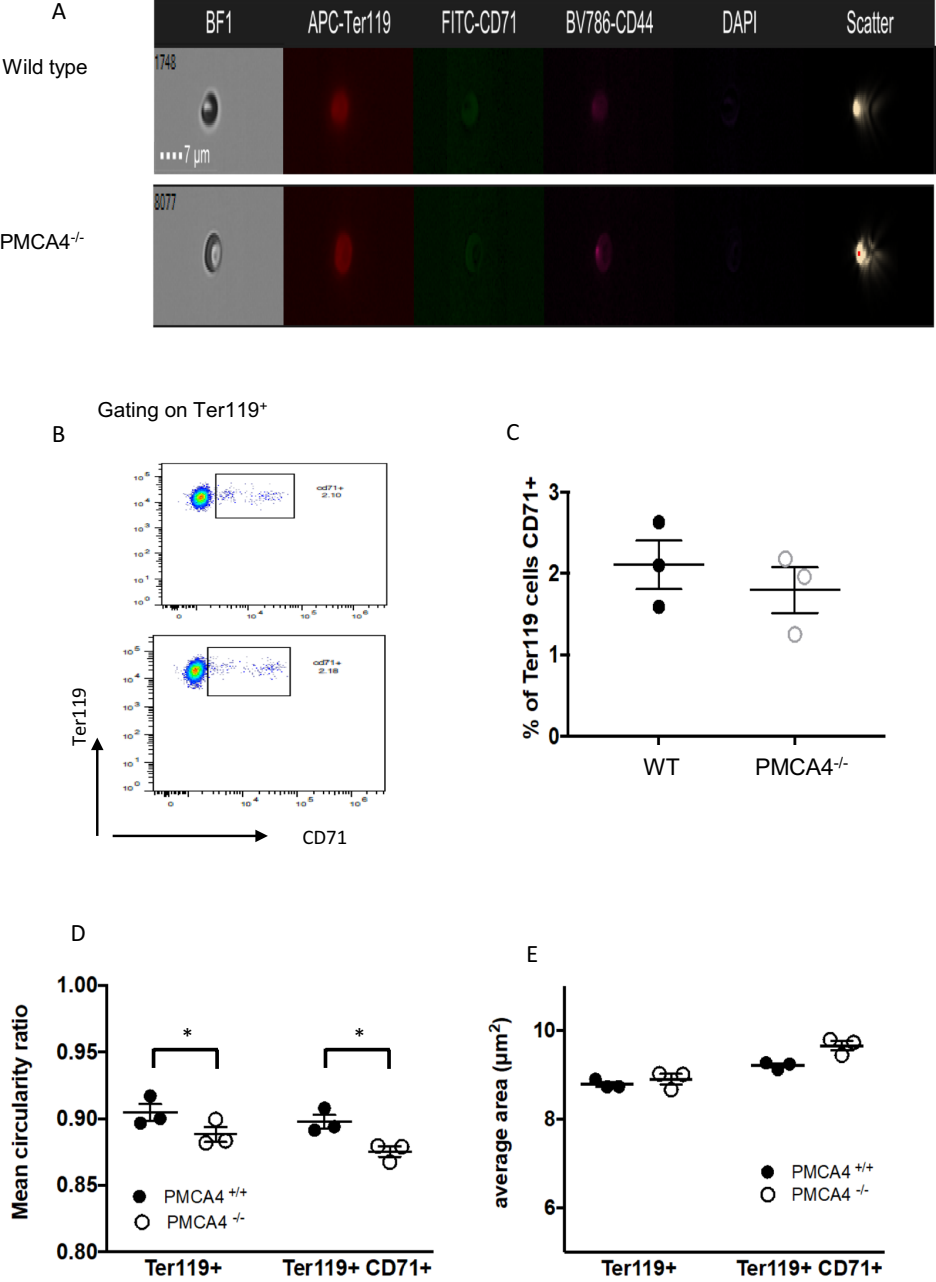
Image stream analysis to assess RBC maturation. Blood was obtained from uninfected PMCA4^−/−^ and wild type (WT) littermates. **A** Representative image stream pictures showing RBC morphology and Ter119 (pan RBC), CD71 (reticulocyte) and CD44 (early erythroblast) expression and **B** representative dot plot showing CD71 expression by Ter119^+^ RBCs. **C** Percentage of Ter119^+^ RBCs that are CD71^+^ reticulocytes. **D** Mean circularity ratio and **E** average area of Ter119^+^ mature RBCs and Ter119^+^CD71^+^ reticulocytes (N = 3 in each group, *P < 0.05)

There is evidence that the maturation programme of red blood cells, specifically immature reticulocytes, is influenced by intracellular calcium concentrations [21]. Therefore, image stream analysis was performed to investigate if PMCA4 deletion influenced the maturation state of red blood cells (Fig. 2A, B). Surprisingly, there was no difference in the frequencies of reticulocytes (CD71^+^) within the RBC (Ter119^+^) population between PMCA4^−/−^ and WT mice (Fig. 2C). It is also important to note that there were only very few early erythroblasts (CD44^+^ cells) observed in the analyses (results not shown). The image stream analysis did show that the mean circularity ratio of mature Ter119^+^ normocytes and CD71^+^ reticulocytes was slightly lower in PMCA4^−/−^ mice, suggesting that PMCA4 deficiency affected RBC morphology (Fig. 2D). In contrast to the results from the automatic blood analyser, the image stream analysis did not reveal any major differences in the size of mature normocytes or immature reticulocytes in the PMCA4^−/−^ mice, compared with control WT mice (Fig. 2E).

Combined, these data indicate that the deletion of PMCA4 had an effect in modulating the haemoglobin content of RBCs (an important determinant that can influence *Plasmodium* growth and survival within RBCs), and potentially had a small impact on RBC size.

### Deletion of PMCA4 does not affect the course of blood-stage *Plasmodium chabaudi* infection

**Fig. 3:**
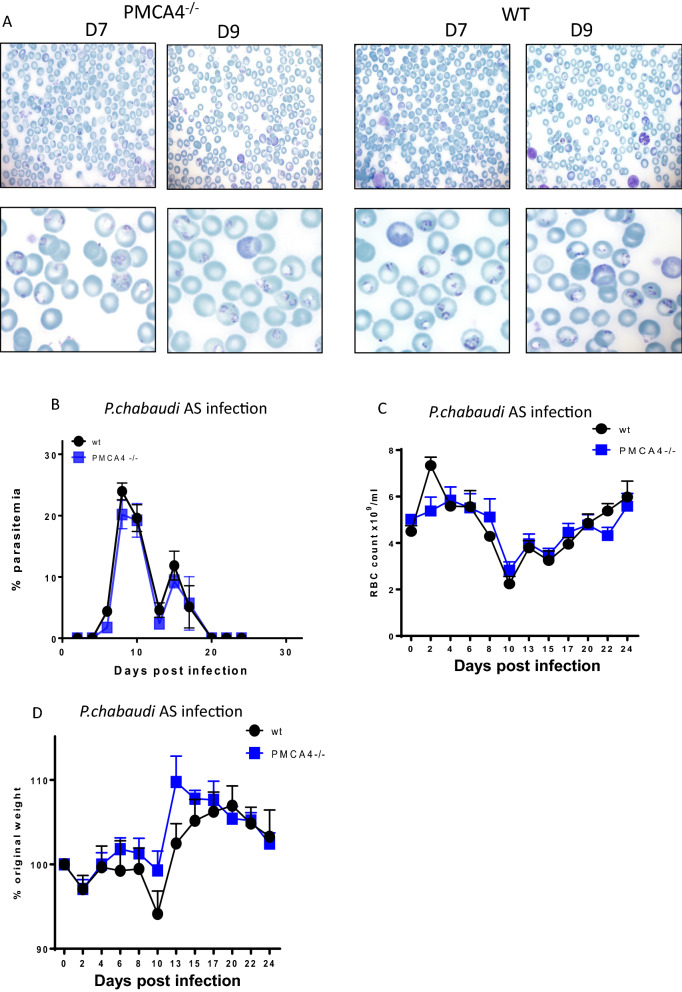
The course of *P. chabaudi* AS infection in PMCA4^−/−^ mice. PMCA4^−/−^ and WT littermate mice were infected (i.v.) with 10^4^ *P. chabaudi* AS pRBCs. **A** Representative pictures of Giemsa-stained thin blood smears showing RBCs and pRBCs from days 7 and 9 of infection. **B**–**D** The course of infection as measured by **B** peripheral parasitaemia. **C** RBC counts and **D** change in body weight. The results are from one of three independent experiments with n = 3–5 per group, in each independent experiment

To investigate how systemic abrogation of PMCA4 expression, and the associated alterations in RBC properties, affected the course of blood-stage malaria, the PMCA4^−/−^ and WT littermate mice were infected with *P. chabaudi* parasites, a species of rodent *Plasmodium* that predominantly infects mature erythrocytes (normocytes) and which causes recrudescing and chronic malarial disease [22, 23]. Unexpectedly, there were no qualitative differences in the morphology (diameter or shape) of infected RBCs in PMCA4^−/−^ and WT mice (as shown on day 7 and 9 infection) (Fig. 3A). Moreover, there were no obvious qualitative differences in the proportions, or phenotype, of early ring stage, trophozoite or schizont intraerythrocytic stages of the parasite, or abundance of extracellular parasites, in PMCA4^−/−^ and WT littermate mice (Fig. 3A). In agreement, the course of *P. chabaudi* infection was comparable in PMCA4^−/−^ and littermate WT mice, with no difference in level of peak parasitaemia on day 9 of infection and equivalent size of recrudescence, which was cleared at equal rate, in PMCA4^−/−^ and WT mice (Fig. 3B). Correspondingly, there were no differences in signs of disease morbidity (reduction in RBCs per ml or weight) between PMCA4^−/−^ and WT mice during the course of *P. chabaudi* infection (Fig. 3C, D). Moreover, computational image analysis of Giemsa-stained thin blood smears suggested that there were no marked differences in the radius ratio, circumference (perimeter), diameter and circularity of RBCs during the course of *P. chabaudi* infection between PMCA4^−/−^ and WT mice (Additional file 1: Fig. S1A–D). Analysis of reticulocyte level also showed no difference between PMCA4^−/−^ and WT mice during *P. chabaudi* infection (Additional file 1: Fig. S1E). Thus, surprisingly, genetic deletion of PMCA4 did not significantly influence the disease severity or level of peripheral parasitaemia during *P. chabaudi* AS infection.

### Deletion of PMCA4 does not affect the course of reticulocyte-restricted *Plasmodium yoelii* infection

**Fig. 4:**
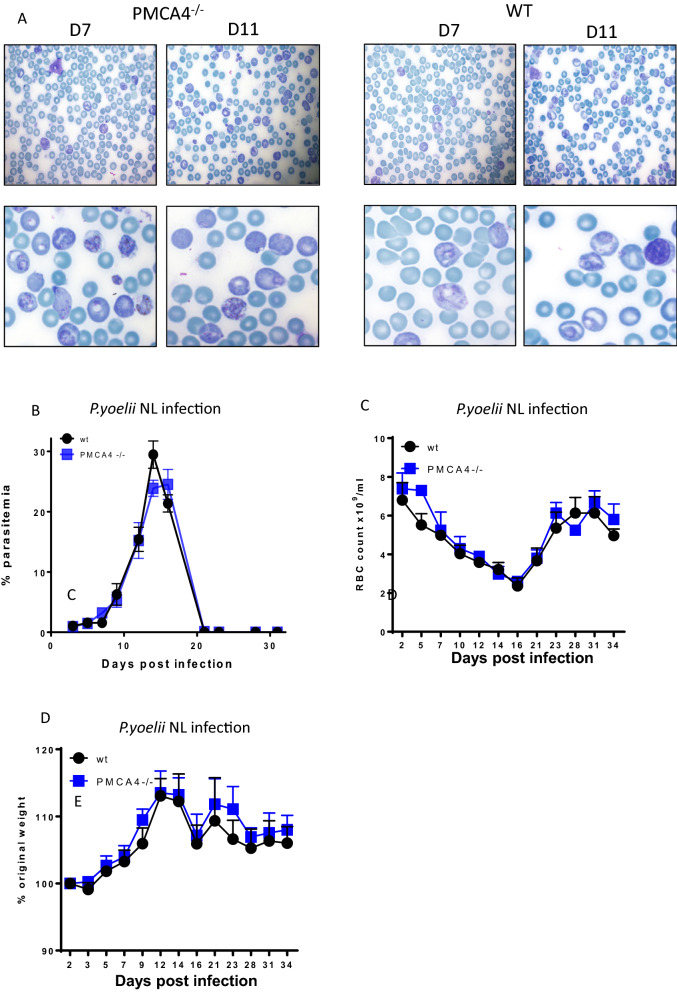
The course of *P. yoelii* NL infection in PMCA4^−/−^ mice. PMCA4^−/−^ and WT littermate mice were infected (i.v.) with 10^4^ *P. yoelii* NL pRBCs. **A** Representative pictures of Giemsa-stained thin blood smears showing RBCs and pRBCs from days 7 and 11 of infection. The course of infection as measured by **B** peripheral parasitaemia, **C** RBC counts and (**D**) change in body weight. The results are from one of three independent experiments with n = 3–5 per group, in each independent experiment

Abrogation of PMCA4 did not affect the course of *P. chabaudi* infection, in which the parasite primarily invades mature erythrocytes. *Plasmodium* parasites have differing requirements for growth and survival in reticulocytes than in mature erythrocytes [24] and calcium levels are differentially controlled in reticulocytes than in normocytes [21, 25]. Therefore, it is important to investigate if the absence of PMCA4 affected the course of infection caused by a reticulocyte-restricted parasite, *P. yoelii*, which typically causes a gradual and self-resolving blood-stage malaria [26]. As expected, *P. yoelii* parasites exhibited a strong preference for reticulocytes (Fig. 4A). There were no qualitative differences in the extent of restriction of parasites towards reticulocytes in PMCA4^−/−^ and WT mice, as shown on days 7 and 11 of infection (Fig. 4A). The morphology of infected reticulocytes were also unaltered in PMCA4^−/−^ and WT mice, and the relative proportions and phenotype of the different intra-erythrocytic life-cycle stages were qualitatively comparable in PMCA4^−/−^ mice compared with WT mice (Fig. 4A). Accordingly, there were no significant differences in the course of *P. yoelii* infection (as determined by peripheral parasite levels, RBC/ml counts and percentage weight change), in PMCA4^−/−^ mice compared with WT mice (Fig. 4B–D). Similar to the finding in *P. chabaudi AS* model, there were no significant changes in the radius ratio, circumference (perimeter), diameter and circularity of RBCs during the course of *P. yoelii* infection between PMCA4^−/−^ and WT mice (Additional file 1: Fig. S2A–D). Reticulocyte level, as indicated by analysis on Giemsa-stained blood smear, was not different between PMCA4^−/−^ and WT mice during *P. yoelii* infection (Additional file 1: Fig. S2E). Thus, knock out of PMCA4 did not affect the dynamics or severity of reticulocyte-restricted blood-stage malaria in mice.

### Deletion of PMCA4 provides partial protection against experimental cerebral malaria

**Fig. 5:**
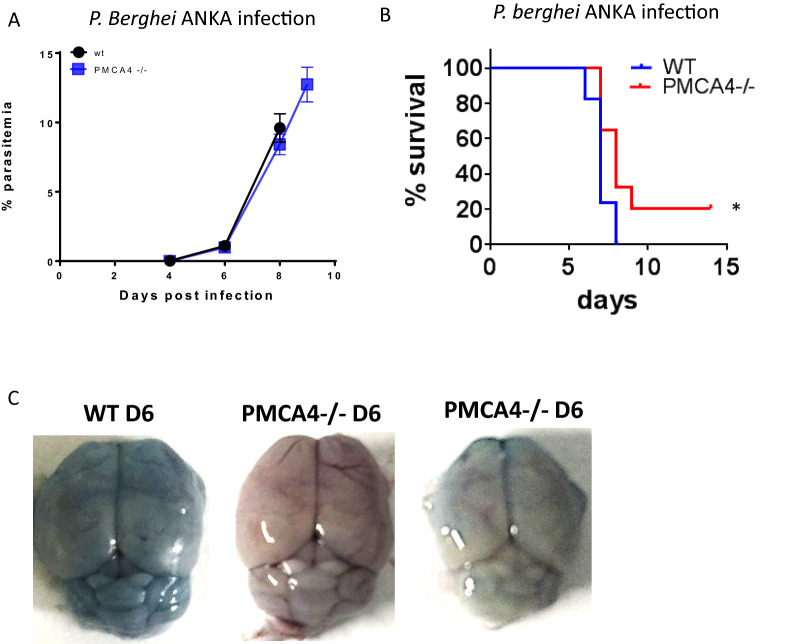
The development of ECM in PMCA4^−/−^ mice during *P. berghei* ANKA infection. PMCA-4^−/−^ and WT littermate mice were infected (i.v.) with 10^4^ *P. berghei* ANKA pRBCs. **A**, **B** The course of infection as measured by (**A**) peripheral parasitaemia and **B** survival. **C** Representative pictures showing level of Evans blue penetration into brain on day 6 of infection (Evans blue injected (i.v.) 1 h prior to removal of brain). **A** The results are from one of three independent experiments with n = 3–5 per group, per experiment. **B** The data includes all mice used in the project (n = 34 per group, * P < 0.05)

The PMCA4 gene is an identified resistance locus for cerebral malaria, the development of which is often poorly correlated with circulating parasite levels [27]. Thus, whilst the above results suggested that PMCA4 did not significantly influence peripheral parasitaemia and the course of mild murine blood-stage malarias, this did not rule out a role for PMCA4 in influencing severe malarial disease. To address this, the development of ECM in PMCA4^−/−^ mice during blood-stage *P. berghei* infection was assessed. As expected, PMCA4^−/−^ mice and WT littermate mice exhibited equivalent peripheral parasite burdens during *P. berghei* infection (Fig. 5A). However, PMCA4^−/−^ mice were partially resistance to development of ECM, with 20% of PMCA4 KO mice (compared with 0% of WT littermate mice) surviving the window period of ECM development (day 6–10) (Fig. 5B). In agreement, on day 6 of infection, when WT mice began showing signs of ECM, PMCA4^−/−^ mice showed qualitatively variable, but generally lower, levels of Evans blue staining within the brain compared with WT littermate mice (Fig. 5C). This qualitative assessment indicates that the absence of PMCA4 variably reduced and /or delayed blood brain barrier disruption during *P.berghei* infection, consistent with the staggered kinetics of ECM and the resistance of a small proportion of PMCA4^−/−^ mice to the syndrome.

**Fig. 6:**
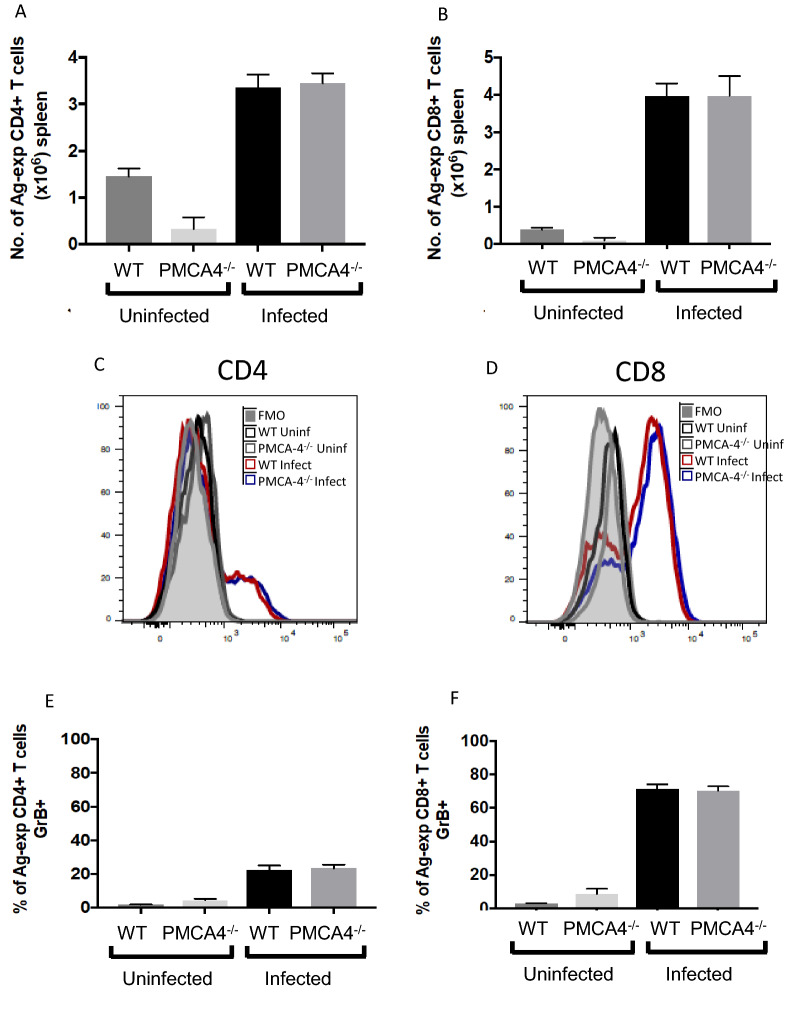
The effect of PMCA4 ablation on splenic immune responses during *P. berghei* ANKA infection. PMCA4^−/−^ and WT littermate mice were infected (i.v.) with 10^4^ *P. berghei* ANKA pRBCs. **A**, **B** The numbers of antigen-experienced (Ag-exp: CD11a^+^CD49d^+^) **A** CD4^+^ T cells and **B** CD8^+^ T cells in the spleen on day 6 of infection. **C**, **D** Representative histograms and percentages of splenic **E** Ag-exp CD4^+^ T cells and **F** Ag-exp CD8^+^ T cells expressing Granzyme B on day 6 of infection. (Data are from one of two independent experiments with n = 4–5 per group, per experiment)

**Fig. 7:**
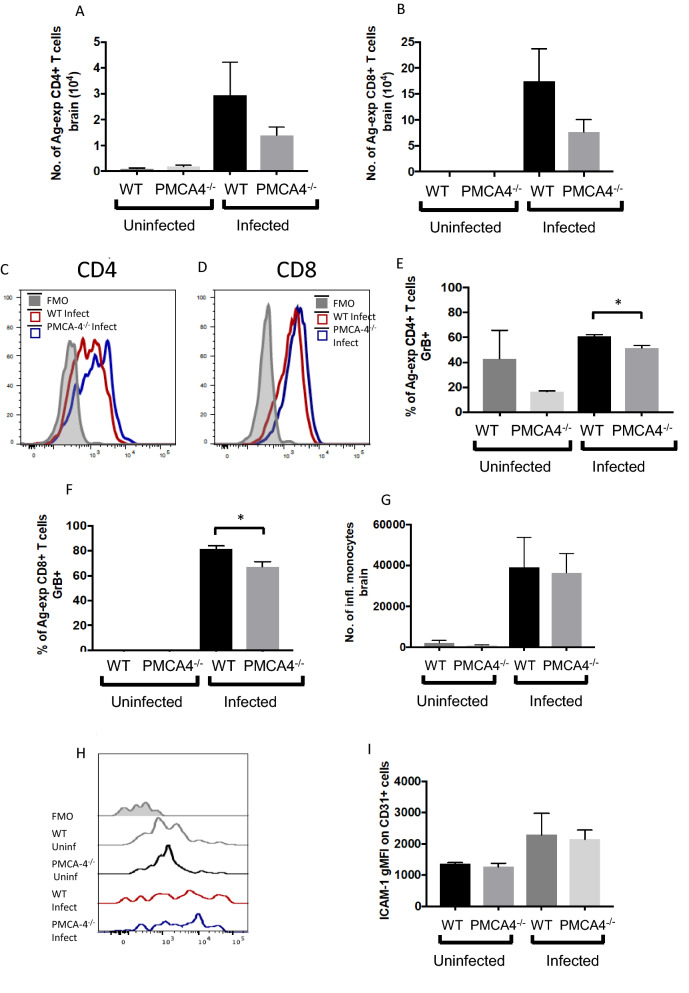
Analysis of cerebral T-cells during *P. berghei* infection. PMCA4^−/−^ and WT littermate mice were infected (i.v.) with 10^4^ *P. berghei* pRBCs. **A**, **B** The numbers of **A** Ag-exp CD4^+^ T cells and **B** Ag-exp CD8^+^ T cells in the brain on day 6 of infection. **C**, **D** Representative histograms and percentages of intracerebral **E** Ag-exp CD4^+^ T cells and **F** Ag-exp CD8^+^ T cells expressing Granzyme B on day 6 of infection. **G** The numbers of inflammatory (Ly6c^+^) monocytes in the brain on day 6 of infection. **H** and **I** Representative histograms and percentages of CD31^+^CD45^−^ endothelial cells expressing ICAM-1 on day 6 of infection. The results are from one of two independent experiments with n = 4–5 per group, per experiment (* p < 0.05)

ECM is a T cell-dependent syndrome, where T cells undergo activation in the spleen before migrating to the brain where they interact with endothelial cells lining the cerebrovasculature, causing blood brain barrier damage through Granzyme B production [28–31]. Thus, it is important to examine if the partial resistance of PMCA4^−/−^ mice to ECM was associated with alterations in the splenic and/or intracerebral T cell response. Abrogation of PMCA4 did not significantly alter the numbers of activated (antigen-experienced) CD4^+^ and CD8^+^ T cells (as defined by upregulation and co-expression of CD11a and CD49d [32, 33];) in the spleen on day 7 of infection. Nor did deletion of PMCA4 affect the percentages of antigen-experienced splenic CD4^+^ T cells or CD8^+^ T cells expressing Granzyme B (Fig. 6A-F). However, the numbers of antigen-experienced T cells accumulating in the brain trended lower in PMCA4^−/−^ mice on day 7 of infection (compared with WT littermate mice) and intracerebral antigen-experienced CD4^+^ and CD8^+^ T cells expressed slightly (but significantly) lower levels of GrB in infected PMCA4^−/−^ mice than in WT littermate mice (Fig. 7A–F, with gating strategy as shown in Additional file 1: Fig. S1). Of note, there were no differences in microglial numbers or neutrophil or monocyte accumulation within the brains of PMCA4^−/−^ and WT mice on day 7 of infection (Fig. 7G, including Additional file 1: Fig. S1). Moreover, ICAM-1 expression by brain CD31^+^ cells (putative endothelial cells), was also comparable in infected PMCA4^−/−^ and WT mice on day 7 of infection (Fig. 7H–I). Thus, the partial resistance of PMCA4^−/−^ mice to ECM was associated with a slight reduction in the strength of pathogenic intracerebral T cell response, rather than a generalized dampening of cerebral inflammation.

## Discussion

The key finding of our study is that genetic ablation of PMCA4 expression in mice slightly improved the survival against *P. berghei* infection, which is a common murine model of cerebral malaria. However, PMCA4 deficiency did not alter the course of non-lethal malaria during *P. chabaudi* and *P. yoelii* infections. Moreover, the deletion of PMCA4 did not impact peripheral parasite levels during any of the murine malaria models examined.

The PMCA4 SNPs that have been associated with risk to severe malarial disease are believed to control the level of PMCA4 expressed on RBCs [34]. Specifically, PMCA4 SNPs that correspond with reduced PMCA4 expression (and consequent increased intracellular Ca^2+^ levels) appear to be associated with protection against severe malaria [34]. PMCA4-deficiency in red blood cells has previously been shown to lead to increased mean corpuscular haemoglobin concentration (indicating reduced RBC hydration), likely due to Ca-induced activation of potassium channel resulting in potassium efflux and volume loss (the Gardos effect) [13]. The hydration state of RBCs has been demonstrated to strongly influence the invasion and growth of *Plasmodium* parasites, with *P. falciparum* parasites being unable to invade dehydrated cells, potentially due to membrane-cytoskeletal alterations precluding correct orientation of merozoites [14, 35]. Thus, it was very surprising that there did not appear to be any obvious differences in the status of intra-erythrocytic parasites, or the morphology of parasitized RBCs, in PMCA4^−/−^ mice compared with WT mice, and that peripheral parasitaemia levels were not altered in PMCA4^−/−^ mice during any of the investigated murine *Plasmodium* spp. infections within this study.

Whilst the above analyses did show differences in the mean corpuscular haemoglobin levels in RBCs from PMCA4^−/−^ mice, the majority of other analysed RBC properties (size, morphology, maturation) were only minorly affected or were unaffected by abrogation of PMCA4 expression. Indeed, whilst the results from the automated blood analyser suggested that mean corpuscular volume of RBCs was lower in PMCA4^−/−^ mice, this was not recapitulated within the image stream analysis. There were also no obvious signs of increased proportions of crenated and dehydrated RBCs, or difference in RBC diameter, perimeter and circulaty in infected PMCA4^−/−^ mice when studying Giemsa-stained thin blood smears. Thus, the data suggests that abrogation of PMCA4 in murine RBCs may not perturb RBC properties to a sufficient level to affect *Plasmodium* parasite invasion, growth or survival. Indeed, the effect of RBC dehydration on parasite invasion and growth appears to be a graded phenomenon that only becomes significant above a threshold level of dehydration [14]. The finding of this study, therefore, appears to question the dominant impact of PMCA4 in controlling *Plasmodium* invasion, growth or survival within RBCs (normocytes or reticulocytes) during murine malaria.

It is important to note that it has yet to be definitively shown in any study that PMCA4 directly affects *Plasmodium* parasite growth in RBCs. To directly test this question it will be necessary to isolate RBCs from individuals with different PMCA4 SNPs and culture them in vitro with *P. falciparum* parasites. As intracellular Ca^2+^ levels dictate many different biophysical properties of RBCs [36], the possibility that alterations in PMCA4 expression level affect other pRBC properties cannot be discounted. This includes several pRBCs properties, such as transport and membrane expression of *Plasmodium* proteins and cellular deformability, that could influence the development of severe malaria, and which may feature more prominently in human blood-stage malaria than murine malaria. Moreover, in extreme cases, alterations in the morphology of RBCs may lead to increased phagocytosis within the spleen by red pulp macrophages, which clear damaged and old RBCs from circulation.

Several studies have shown that some genetic conditions involving haemolytic anaemia (including thalassemia and sickle cell disease) are often associated with “leakiness” of the RBC membrane, which lead to the increase in intracellular Ca^2+^ level [36]. However, other reported observations have shown that the mechanisms underlying protection against malaria in these conditions were reduction in haemoglobin level [37] and reduction in parasite cytoadherence [38], but not due to the increase in RBCs’ Ca^2+^ level. This is in line with the finding in PMCA4^−/−^ mice that the levels of parasitaemia following *P. chabaudi* and *P. yoelii* infection were not different between WT and PMCA4^−/−^ mice.

Although the focus of the present study was on the potential role of PMCA4 on RBCs in influencing the course of blood stage malaria, the PMCA isoforms 1 and 4 are ubiquitously expressed in most organs and cell types [9]. Interestingly, PMCAs may have different roles and functions in different cell types. For example, in vascular smooth muscle cells and in cardiac myocytes PMCA4 appears to modulate cellular signalling, including regulating nitric oxide (NO) production by neuronal nitric oxide synthase (nNOS)[39–41]. In endothelial cells, PMCA4 modulates vascular endothelial growth factor (VEGF)-induced angiogenesis via regulation of calcineurin [42]. In fibroblast cell line L929, PMCA4 is involved in regulating signalling pathways controlling responsiveness to tumour necrosis factor (TNF)[43], whilst in T-cells, PMCA4 is involved in the production of inflammatory cytokines, such as interleukin-2 (IL-2) [44]. Endothelial cells and various leucocyte populations including macrophages, neutrophils and T cells, play important roles in the pathogenesis of severe malarial disease syndromes, including cerebral malaria. Consequently, it is foreseeable that the association of PMCA4 SNPs with risk of severe malaria is not solely RBC-dependent, but is multifactorial, involving modulation of host pro-inflammatory immune responses and control of vascular homeostasis. In support of this, we observed qualitatively lower damage to the blood brain barrier in PMCA4^−/−^ mice compared with control mice during *P. berghei* infection, corresponding with the slightly increased resistance of PMCA4^−/−^ mice to ECM. In addition, there was a trend towards lower numbers of T cells in the brains of PMCA4^−/−^ mice during *Plasmodium berghei* infection, and intracerebral T cells that did accumulate also expressed lower levels of Granzyme B in infected PMCA4^−/−^ mice. Although the IFN-g production by brain accumulating CD8^+^ T cells was not assessed, it can be anticipated that the level of this cytokine may also have been lower within infected PMCA4^−/−^ mice than in infected control WT mice, given that Granzyme B and IFN-g are sensitive measures of CD8^+^ T cell pathogenic activity during *P. berghei* infection [30]. Notably, however, the numbers of monocytes, neutrophils and resident microglial cells within the brain were not significantly altered in PMCA4^−/−^ mice compared with control WT mice during *P. berghei* infection. This suggests that global PMCA4 deletion did not lead to a generalized amelioration of neuroinflammation during *P. berghei* infection. Thus, further work will be required to assess whether the specific alterations in T cell activity in the brain of infected PMCA4^−/−^ mice, and the associated slight increase in resistance to ECM, was due to cell autonomous effects of PMCA4 deficiency or was caused by other discrete indirect effects within the brain. Indirect effects that may influence CD8^+^ T cell responses in the brain during *P. berghei* ANKA infection could potentially include reduced pRBC accumulation and cross presentation of parasite antigen by brain endothelial cells, or alterations in CXCL9 and CXCL10 production, which are critical events in the pathogenesis of ECM [45–48].

## Conclusions

Studies using PMCA4^−/−^ mice indicate that although PMCA4 ablation in RBC does not seem to affect *Plasmodium* life cycle in RBCs, PMCA4 may be involved, at least in part, in the development of cerebral malaria.

## Supplementary Information

**Additional file 1.** Additional figures and tables.

## Data Availability

All data generated or analysed during this study are included in this published article and its additional information files.

## Abbreviations

PMCA: Plasma membrane calcium ATPase
RBCs: Red blood cells
GWAS: Genome wide association study
SNPs: Single nucleotide polymorphisms
ECMs: Experimental cerebral malaria
MCV: Mean corpuscular volume
MCH: Mean corpuscular haemoglobin
nNOS: Neuronal nitric oxide synthase
VEGF: Vascular endothelial growth factor
TNF-α: Tumour necrosis factor alpha
IL-2: Interleukin-2
