# Supplementary material for: The plasma membrane calcium ATPase 4 does not influence parasite levels but partially promotes experimental cerebral malaria during murine blood stage malaria
Source: Malar J. 2021 Jul 2;20:297. doi: 10.1186/s12936-021-03832-w (PMC8252299; doi:10.1186/s12936-021-03832-w)
Supplement: Supplementary file 1 — Additional file 1. Additional figures and tables. [file 12936_2021_3832_MOESM1_ESM.pdf]

Supplementary Figures

The calcium pump ATPase 4 does not influence parasite levels but partially promotes experimental cerebral malaria during murine blood stage malaria

Ana Villegas-Mendez[1], Nicholas Stafford[2], Michael J. Haley[1], Normalita Eka Pravitasari[3], Florence Baudoin[2], Adnan Ali[4,5], Puji Budi Setia Asih[3], Josephine E. Siregar[3], Esther Baena[4], Din Syafruddin[3], Kevin N. Couper[1,*], Delvac Oceandy[2,*]

Running title: PMCA4 ablation in malaria infection

Supplementary figure 1

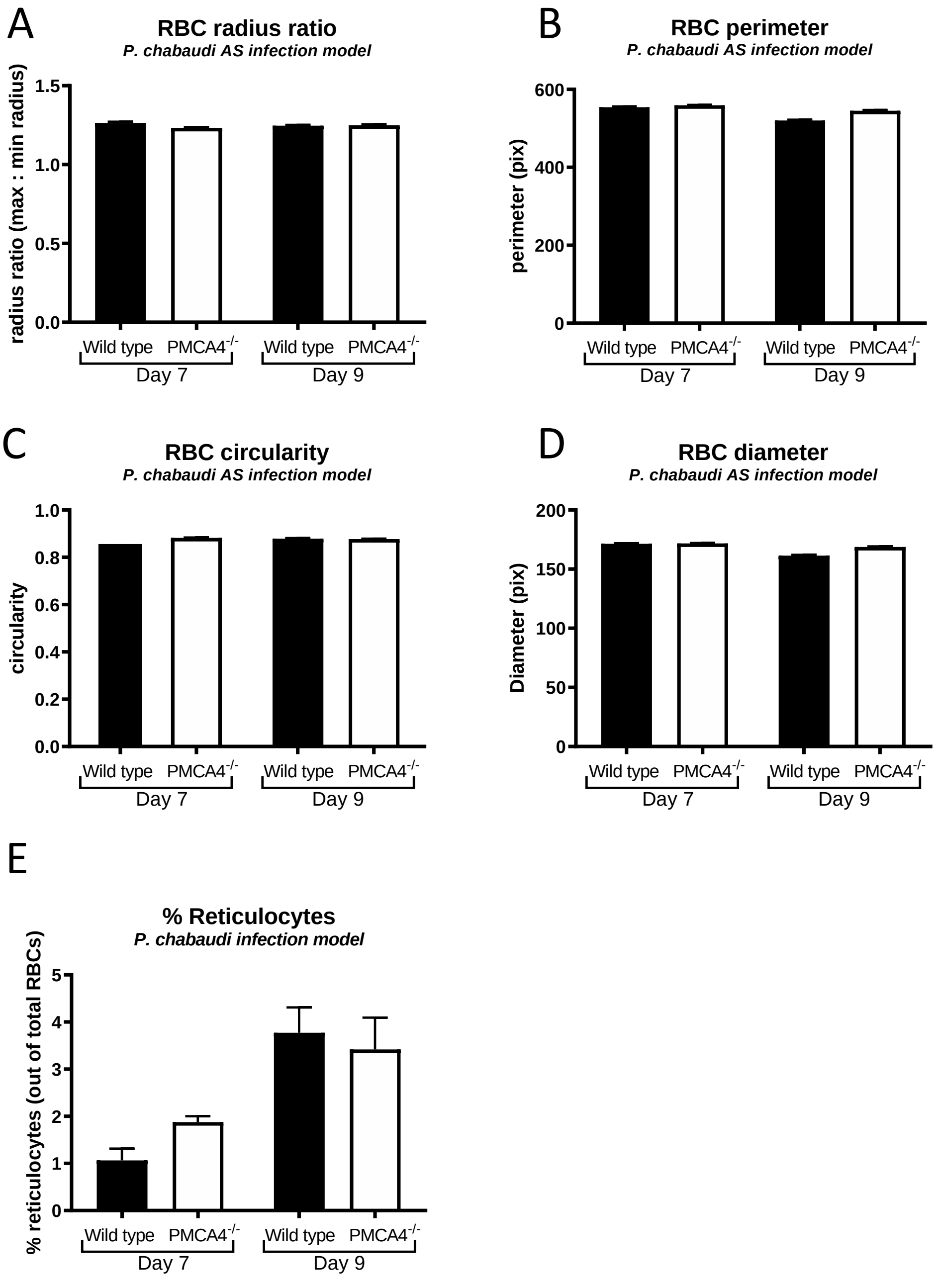

Supplementary Figure 1: Analysis of RBCs during *P. chabaudi* AS infection. RBCs of the Giemsa-stained thin blood smears from WT and PMCA4$^{-/-}$ mice on days 7 and 9 of *P. chabaudi* AS infection were analysed using Image Pro Premier image analysis software. There was no marked difference on **A)** RBC radius ratio, **B)** RBC perimeter, **C)** RBC circularity and **D)** RBC diameter between WT and PMCA4$^{-/-}$ mice at days 7 and 9 of *P. chabaudi* AS infection. **E)** Relative frequency of reticulocytes was analysed on the Giemsa-stained blood smears. No difference in reticulocytes proportion between WT and PMCA4$^{-/-}$ mice at days 7 and 9 following *P. chabaudi* AS infection. A total of 38 fields of view from 3 mice per group per time point was analysed.

Supplementary figure 2

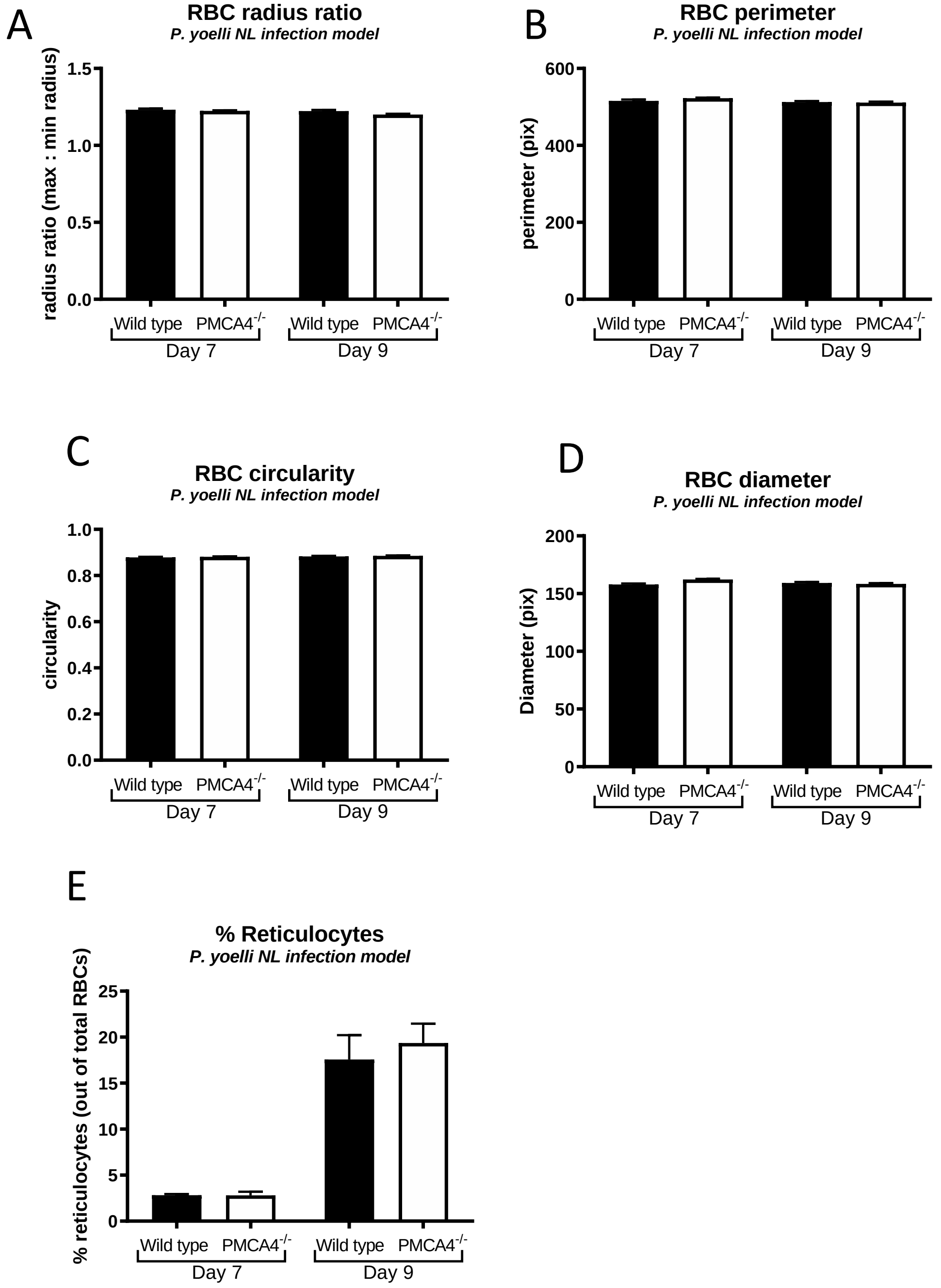

Supplementary Figure 2: Analysis of RBCs during *P. yoelii* NL infection. Computational analysis of RBCs showed that there was no marked difference on **A)** RBC radius ratio, **B)** RBC perimeter, **C)** RBC circularity and **D)** RBC diameter between WT and PMCA4$^{-/-}$ mice at days 7 and 9 of *P. yoelii* NL infection. **E)** Consistent with the data from *P.chabaudi* AS model there was no difference in reticulocytes proportion between WT and PMCA4$^{-/-}$ mice at days 7 and 9 following *P. yoelii* NL infection. A total of 38 fields of view from 3 mice per group per time point was analysed.

Supplementary figure 3

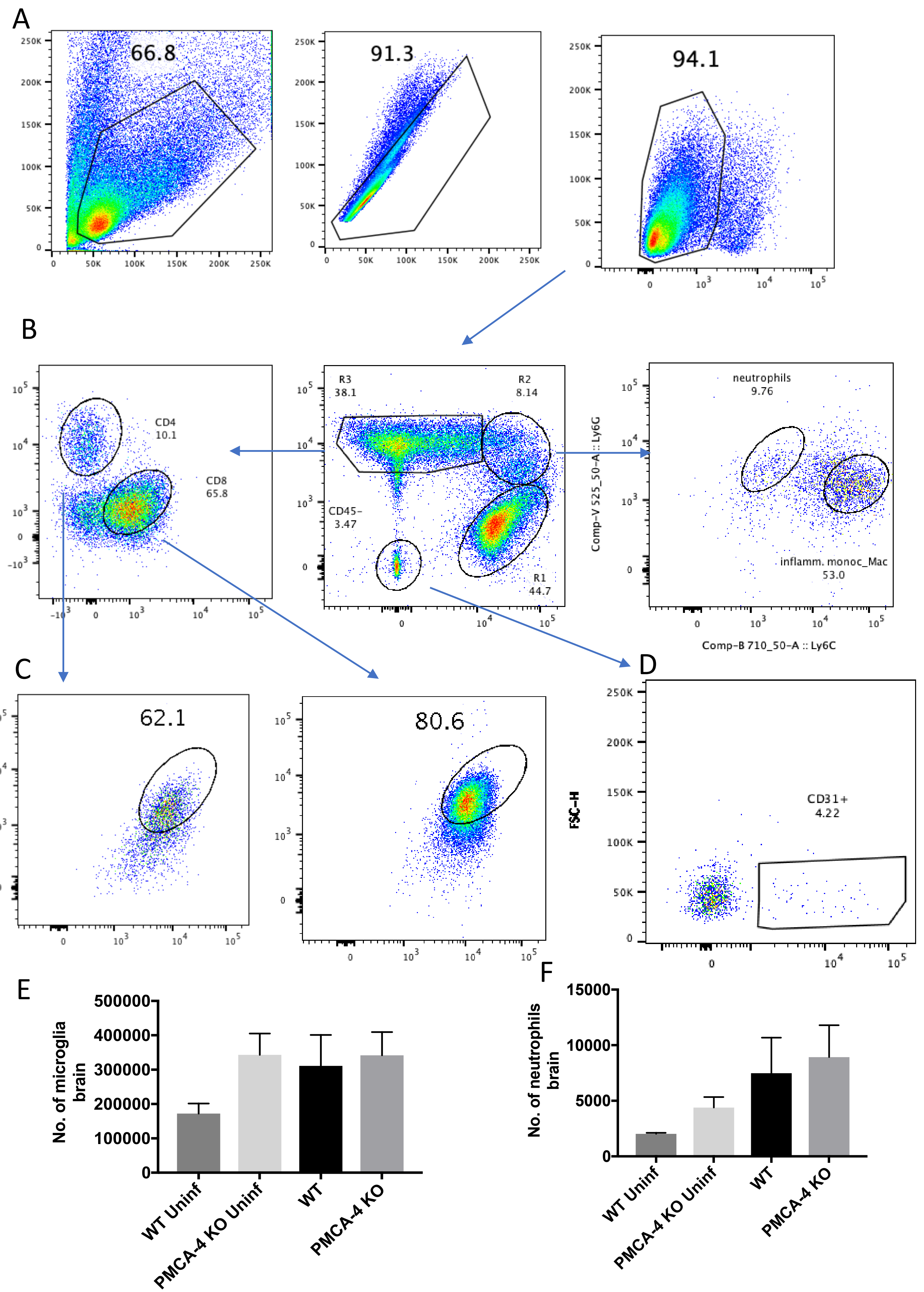

Supplementary Figure 3. The gating strategy for cells within the brain.
Representative flow cytometry plots showing in order: (A) gating of single cells, singlets and live cells. (B) Gating of $CD45^{+}CD11b^{-}$ (R3) populations, $CD45^{+}CD11b^{+}$ (R2) populations, $CD45^{low}$, $CD11b^{+}$ (R1) microglial cells and $CD45^{-}CD11b^{-}$ cells. R2 cells were gated into $Ly6G^{+}Ly6C^{low}$ Neutrophils and $Ly6C^{+}Ly6G^{low}$ inflammatory monocytes. R3 cells were gated into $CD4^{+}$ and $CD8^{+}$ cells. (C) Granzyme B expression by $CD4^{+}$ and $CD8^{+}$ cells. (D) Identification of $CD31^{+}$ endothelial cells within $CD45^{-}CD11b^{-}$ population. (E, F) The calculated numbers of microglia and neutrophils within the brain in uninfected WT and PMCA-4 KO mice and mice infected with 10^{4} *P. berghei* ANKA pRBCs (analysis on day 6 of infection).
